# Long non-coding RNA KCNQ1OT1 overexpression promotes osteogenic differentiation of staphylococcus aureus-infected human bone mesenchymal stem cells by sponging microRNA miR-29b-3p

**DOI:** 10.1080/21655979.2022.2037898

**Published:** 2022-02-28

**Authors:** Ran Ding, Shijun Wei, Ming Huang

**Affiliations:** Department of Orthopedic Surgery, Wuhan General Hospital of People’s Liberation Army, Wuhan City, China

**Keywords:** KCNQ1OT1, miR-29b-3p, osteogenic differentiation, staphylococcus aureus, osteomyelitis

## Abstract

Osteomyelitis (OM) is an orthopedic disease caused by bone infections in the bone cortex, bone marrow, periosteum, and surrounding soft tissues. Recent studies have implicated non-coding RNAs (ncRNAs) in the development of OM. However, little is known about the role of ncRNAs in the osteogenic differentiation during bone infection. In the present study, we investigated the role of KCNQ1OT1/miR-29b-3p axis in osteogenic differentiation in staphylococcus aureus (SpA)-infected human bone mesenchymal stem cells (hBMSCs). We first examined the expression of lncRNA KCNQ1OT1 and miR-29b-3p in the serum samples of OM patients and healthy controls. We also infected hBMSCs with different concentrations of SpA and studied the osteogenic differentiation after infection. Our results revealed that KCNQ1OT1 was downregulated while miR-29b-3p was upregulated in the serum samples of OM patients, as well as in SpA-infected hBMSCs. Overexpression of KCNQ1OT1 ameliorated the damage in hBMSCs caused by SpA infection. KCNQ1OT1 could support hBMSCs osteogenic differentiation by enhancing ALP activity, alizarin red S accumulation, expressions of osteogenic markers, and attenuating inflammatory responses after SpA infection. We further showed that miR-29b-3p was a downstream target of KCNQ1OT1, mediating the osteogenic differentiation of hBMSCs during SpA infection. Our data suggest that KCNQ1OT1 could ameliorate the SpA-induced suppression of osteogenic differentiation in hBMSCs by sponging miR-29b-3p. Modulating KCNQ1OT1 expression may serve as a strategy to ameliorate osteomyelitis.

## GRAPHICAL ABSTRACT

**Figure:**
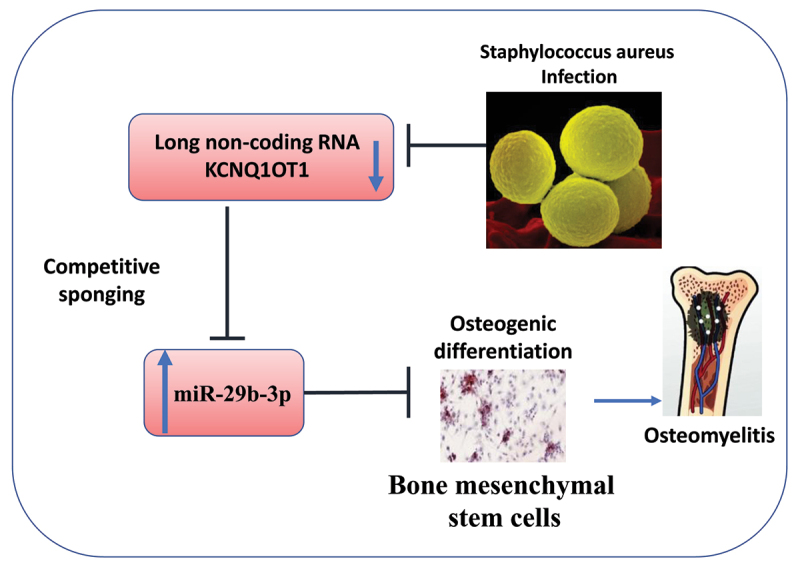

## Introduction

Osteomyelitis (OM) is an orthopedic disease caused by bone infection, which is characterized by serious bone loss and bone destruction [1]. As an inflammatory disease, OM is frequently caused by the infection and invasion of microorganisms in bone tissues, resulting in progressive bone destruction [2]. The remodeling of bone tissues is necessary for the growth and development of skeleton, which is regulated by the dynamic balance between bone resorption and bone formation [3,4]. The maturation of osteoblasts is characterized by the high expression of alkaline phosphatase (ALP), which is an essential enzyme for bone deposition and mineralization [5]. Meanwhile, mature osteoblasts could produce large amounts of osteocalcin (OCN) and osteopontin (OPN) to regulate bone mineralization [6].

Many pathogenic bacteria are associated with OM [7], with the most common microorganism being the gram-positive staphylococcus aureus (SpA). Analysis of clinical specimens from patients with OM showed that SpA accounts for up to 80% of microbial infections [8]. SpA-infected bone tissue display altered adhesive properties and the infection of bone tissue disrupts the dynamic balance between osteoblasts and osteoclasts in the process of bone remodeling [9]. The biofilm formed during the infection process allows the SpA to evade the immune surveillance and boosts the resistance to antibiotics; while the poor local blood supply in the bone lesion makes it difficult to eliminate pathogenic bacteria by drugs or immune cells [10]. Therefore, the treatment and eradication of microbial infection in OM patients remains a challenge in clinical practice.

Non-coding RNAs (ncRNAs) are a class of RNAs without protein-encoding functions, which includes microRNAs (miRNAs), circular RNAs and long non-coding RNAs (lncRNAs) [11]. Previous studies have demonstrated that lncRNAs and miRNAs play critical roles in many biological processes, such as cell proliferation, apoptosis, and differentiation [12,13]. In addition, the roles of lncRNAs and miRNAs in osteogenic differentiation of stem cells have been widely reported. For example, lncRNA PCAT1 promotes osteogenic differentiation of adipose-derived stem cells by regulating miR-145-5p [14]. LncRNA MCF2L-AS1 inhibits osteogenesis of human bone mesenchymal stem cells (hBMSCs) by sponging miR-33a [15]. LncRNA SNHG1/miR-101/DDK1 axis has been implicated in the attenuation of osteogenic differentiation in hBMSCs [16]. However, whether lncRNAs and miRNAs regulates osteogenic differentiation of SpA-infected cells has not been thoroughly investigated.

In the present study, we explored the aberrant expressions of KCNQ1OT1 and miR-29b-3p in the serum samples from OM patients. We further studied the roles of KCNQ1OT1 and miR-29b-3p in osteogenic differentiation of SpA-infected hBMSCs. Our data suggest that KCNQ1OT1 could ameliorate SpA-induced suppression of osteogenic differentiation in hBMSCs by sponging miR-29b-3p, while provides insight into the potential mechanism of SpA-induced bone destruction in OM patients.

## Material and methods

### Specimen collection

This study collected the blood samples from 44 healthy controls and 40 OM patients between December 2015 and November 2019. All subjects signed an informed consent form, and the Ethics Committee of Wuhan General Hospital of People’s Liberation Army approved the experiment. The 40 OM samples included in the analysis was validated for Staphylococcus aureus infection by 16s rRNA sequencing (Supplementary figure S1). For all enrolled individuals, 10 mL venous blood samples were obtained after 8 h fasting, and the samples were preserved in −80°C until use.

### In vitro SpA-infection cell model

Staphylococcus aureus (subsp. aureus Rosenbach, Cat# 25,923, Methicillin resistant (MRSA)) was purchased from American Type Culture Collection (Manassas, Virginia USA) and cultured in BD™ Tryptic Soy Broth (BD Biosciences, New Jersey, USA). Human bone mesenchymal stem cells were purchased from Cyagen (Suzhou, China) and maintained in a human stem cell complete culture medium (HUXMA-90011; Cyagen, Suzhou, China) under the humidified atmosphere with 5% CO_2_ at 37°C.

To mimic the SpA infection in bone marrow in OM patients, hBMSCs were cocultured with different concentrations of SpA (0, 0.5, 1, 10, and 50 μg/mL). In brief, hBMSCs were seeded in 96-well plates at a density of 2000 cells/well, and cultivated in osteogenic differentiation medium Oricel (Cyagen, Suzhou, China) supplemented with different doses of SpA for 14 d.

miR-29b-3p mimic, miR-29b-3p inhibitor, mimics-NC, inhibitor-NC, ad-NC (control vector), and ad-KCNQ1OT1 (vector overexpressing KCNQ1OT1) were designed and acquired from GenePharma (Shanghai, China). As per the vendor’s instructions, cell transfection was conducted with Lipofectamine 2000 reagent (Invitrogen; Thermo Fisher Scientific, Inc.). In 6 well plate, 60% confluent cells were transfected with 50 nM of microRNA mimic or inhibitor or negative controls according to manufacturer’s instruction. Transfected cells were subjected to subsequent analysis 48 hours post-transfection.

### CCK-8 cell proliferation assay

Cells of different treatments were seeded in to a 96-well plate at a density of 1500 cells/well and cultured in a humidified cell culture incubator for 48 h. 10 μL CCK8 reaction solution (Solarbio, Beijing, China) was added to the cell culture and incubated for 1 h in a humidified cell culture incubator. The light absorption value (OD value) in each condition was captured at 450 nm wavelength on a Synergy H1 microplate reader.

### ELISA assay

TNF-α and IL-1β levels in serum samples from OM patients and healthy controls were measured by corresponding commercial ELISA kits. Human TNF-α ELISA kit (kt98069) and human IL-1β ELISA kit (kt98060) were obtained from Sigma-Aldrich (Shanghai, China).

### RT-qPCR analysis

Total RNAs were extracted from serum samples or cells using TRIzol reagent (Invitrogen; Thermo Fisher Scientific) following the manufacturer’s instructions. cDNA was synthesized by cDNA synthesis kit (Bio-Rad, Shanghai, China), and then qPCR analysis was performed on a 7500 real-time PCR system (Applied Biosystems, Foster City, CA) using iQ™ SYBR® Green Supermix reagent (Bio-Rad, Shanghai, China) as per the vendor’s protocol. The thermal cycling conditions included 35 cycles of 94°C for 15s and 62°C for 20s. The relative expression levels of KCNQ1OT1 and miR-29b-3p were normalized to GAPDH or U6 using the 2^−ΔΔCt^ method. All primer sequences were synthesized and purchased from Shanghai Sangon Biotechnology Co., Ltd. (Shanghai, China): KCNQ1OT1 forward, 5′‐AGGGTGACAGTGTTTCATAGGCT‐3′ and reverse, 5′-GAGGCACATTCATTCGTTGGT-3′; miR-29b-3p forward, 5′-ACACTCCAGCTGGGTAGCACCATTTGAAATC-3′ and reverse, 5′-GTAGGCTACTACAGGATTTG-3′; TNF-α forward, 5′-GCATGATCCGCGACGTGGAA-3′ and reverse, 5′-AGATCCATGCCGTTG GCCAG-3′; IL-1β forward, 5′-AAAAGCTTGGTGATGTCTGG-3′ and reverse, 5′-TTTCAACACGCAGGACAGG-3′; U6 forward, 5′-CTCGCTTCGGCAGCACA-3′ and reverse, 5′-AACGCTTCACGAATTTGCGT-3′; GAPDH forward, 5′-GGTATCGTGGAAGGACTCATGAC-3′ and reverse, 5′- ATGCCAGTGAGCTTCCCGTTCAG-3′.

### Western blot assay

Total proteins were extracted using RIPA reagent (Beyotime Biotechnology, Shanghai, China) and the protein concertation was determined by BCA kit (Beyotime Biotechnology, Shanghai, China) as per the instructions. Then, 25 μg of protein was loaded on 10% SDS-PAGE and transferred on PVDF membranes (Millipore, Billerica, MA). After blocking with 5% skimmed milk for 1 h, PVDF membranes were incubated with primary antibodies (rabbit anti-RUNX2, 1:1000, ab236639; rabbit anti-OCN, 1:1000, ab133612; rabbit anti-OSX, 1:1000, ab209484; rabbit anti-OPN, 1:1000, ab8448; rabbit anti-GAPDH, 1:1000, ab9485; all purchased from Abcam, Shanghai, China) overnight at 4°C. The following day, membranes were washed 3 times with TBST buffer for 5 minutes each, and further incubated with secondary antibody (goat anti-rabbit H&L preadsorbed, 1:1000, ab7090; purchased from Abcam, Shanghai, China) for 2 h at room temperature. The protein bands were visualized using an enhanced chemiluminescence kit (Yeasen, Shanghai, China) and photographed on a gel imager system (Bio-Rad, Hercules, CA, United States). GAPDH functioned as the internal control.

### Measurement of ALP activity

ALP detection kit was obtained from Sigma-Aldrich (Shanghai, China). ALP activity assay was conducted using the ALP detection kit according to the manufacturer’s instructions. Total protein concentrations were measured by BCA kit (Beyotime Biotechnology, Shanghai, China), and ALP activity was normalized to the total protein concentrations.

### Alizarin S staining accumulation analysis

hBMSCs with different treatment were inoculated on 12-well plates with a density of 3 × 10^5^ cells/well and differentiated in osteogenic differentiation medium Oricel (Cyagen, Suzhou, China) for 14 d. Cells were fixed with 4% paraformaldehyde for 15 min and then stained with 1% alizarin red S staining solution (ScienCell, USA) for 10 min. Finally, after washing with PBS twice, stained cells were photographed with an optical microscope (Keyence, Shanghai, China). Alizarin red staining in a stained monolayer by acetic acid extraction and neutralization with ammonium hydroxide followed by colorimetric detection at 405 nm.

### Dual-luciferase reporter assay

Wild-type KCNQ1OT1 (KCNQ1OT1-wt) sequence containing the miR-29b-3p binding site was cloned into the luciferase reporter vector pmirGLO (Promega, Madison, WI). To construct mutant KCNQ1OT1 (KCNQ1OT1-mut), the miR-29b-3 binding site was mutated. hBMSCs were then co-transfected with the KCNQ1OT1-mut or KCNQ1OT1-wt reporter, Renilla luciferase (hRlucneo) control vector in the presence of miR-29b-3p mimic or mimic-NC using Lipofectamine 2000 (Invitrogen; Thermo Fisher Scientific, Inc.) according to the vendor’s instructions. 48 h post transfection, the relative luciferase activities were measured using Dual-Luciferase Reporter Assay Kit (Promega, E1910) on a luminescence microplate reader (Infinite 200 PRO; Tecan). The relative firefly luciferase activity in the reporter plasmid was normalized to that of Renilla luciferase (hRlucneo) control vector.

### RNA pull-down assay

The hBMSCs were transfected with 6 µg biotinylated miR-29b-3p (Bio-miR-29b-3p) or control (Bio-miR-NC). After 48 h transfection, the cells were lysed in IP lysis buffer (Beyotime, P0013). 10% of the lysates was saved as the input. The remaining lysate was further incubated with M-280 streptavidin magnetic beads (Sigma-Aldrich, 11205D) at 4°C shaking overnight. A magnetic bar was used to pull down the magnetic beads and associated nucleic acids, then the samples were washed 4 times with IP buffer. Both the input and the elutes from the pull-down were purified with Trizol reagent, and the relative amount of KCNQ1OT1 in each sample was quantified by RT-qPCR.

### Statistical analysis

All data were presented as mean ± standard deviation (SD) in this study and analyzed by SPSS version 18.0 and Prism GraphPad 8.0 software. Significance between two groups was analyzed using student’s t-test, and comparisons among multiple groups were analyzed using one-way ANOVA followed by Tukey’s post-hot test. Spearman correlation analysis was performed to determine the correlation between the expressions of miR-29b-3p and KCNQ1OT1. P < 0.05 was deemed as statistically significant.

## Results

In this study, we delineated the roles of KCNQ1OT1/miR-29b-3p axis in the regulation of osteogenic differentiation upon SpA infection. We first showed that KCNQ1OT1 was downregulated, while miR-29b-3p was upregulated in the serum samples of OM patients, as well as in SpA-infected hBMSCs. Overexpression of KCNQ1OT1 could promote osteogenic differentiation of hBMSCs upon SpA infection. KCNQ1OT1 could support osteogenic differentiation by enhancing ALP activity, alizarin red S accumulation, expressions of osteogenic markers, and attenuating inflammatory responses. miR-29b-3p was identified as a downstream target of KCNQ1OT1, which mediates the osteogenic differentiation of hBMSCs during SpA infection. Our data suggest that KCNQ1OT1 could ameliorate SpA-induced suppression of osteogenic differentiation by sponging miR-29b-3p.

### KCNQ1OT1 downregulation in SpA-infected hBMSCs is associated with impaired osteogenic differentiation

**Figure 1.**
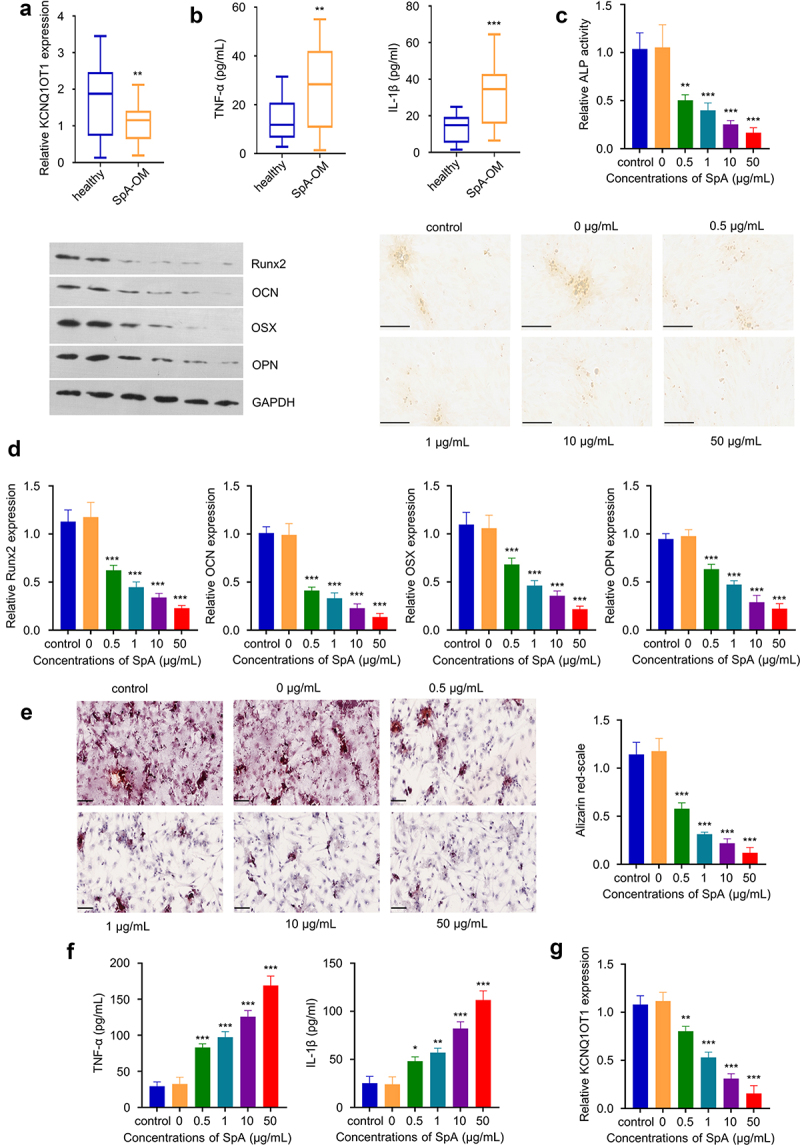
KCNQ1OT1 is significantly down-regulated in SpA-treated hBMSCs. (a) Expression of KCNQ1OT1 in serum samples from OM patients and healthy controls. (b) Expressions of TNF-α and IL-1β in serum samples from OM patients and healthy controls were examined by ELISA assay. (c) ALP activity in hBMSCs treated with different concentrations of SpA after osteogenic differentiation. Scale bar represents 200 μm. (d) Expression levels of osteogenic marker genes in SpA-treated hBMSCs was detected by RT-qPCR and Western blot. (e) Alizarin red S staining in hBMSCs after osteogenic differentiation under different concentrations of SpA. Scale bar represents 100 μm. (f) Expressions of TNF-α and IL-1β in SpA-treated hBMSCs after osteogenic differentiation. (g) KCNQ1OT1 level in hBMSCs treated with different concentrations of SpA after osteogenic differentiation. SpA, staphylococcus aureus; hBMSCs, human bone mesenchymal stem cells; OM, osteomyelitis. *p < 0.05; **p < 0.01; ***p < 0.001.

In serum samples from OM patients, we found KCNQ1OT1 was significantly down-regulated as compared to healthy controls (Figure 1(a)). The 40 OM samples included in the analysis were validated for Staphylococcus aureus (SpA) infection by 16s rRNA sequencing (Supplementary figure 1, see BLAST results). Meanwhile, there was an upregulation of pro-inflammatory cytokines TNF-α and IL-1β in the serum samples of OM patients (Figure 1(b)), indicating an inflammatory response. Furthermore, we infected hBMSCs with different quantities of SpA (0, 0.5, 1, 10 and 50 μg/mL) and then cultured the cells under osteogenic condition. At day 14, ALP activity analysis revealed that SpA infection inhibited ALP activity in dose-dependent manner (Figure 1(c)). Consistently, qRT-PCR and Western blot analysis of osteogenic markers Runx2, OCN, OSX, and OPN showed that SpA infection suppressed the expression of these osteogenic marker genes (Figure 1(d)). Functional analysis by alizarin red S staining further indicates that SpA infection suppressed the cellular mineralization during osteogenic differentiation (Figure 1(e)), with fewer mineralized nodules observed in cells infected with higher amount of SpA. SpA infection also leads to the upregulation of proinflammatory cytokines such as TNF-α and IL-1β (Figure 1(f)), while the expression of KCNQ1OT1 was gradually reduced along with the increasing SpA concentrations in hBMSCs (Figure 1(g)). Together, these data suggest that KCNQ1OT1 downregulation in SpA-infected hBMSCs is associated with impaired osteogenic differentiation.

### KCNQ1OT1 overexpression supports osteogenic differentiation SpA-infected hBMSCs

**Figure 2.**
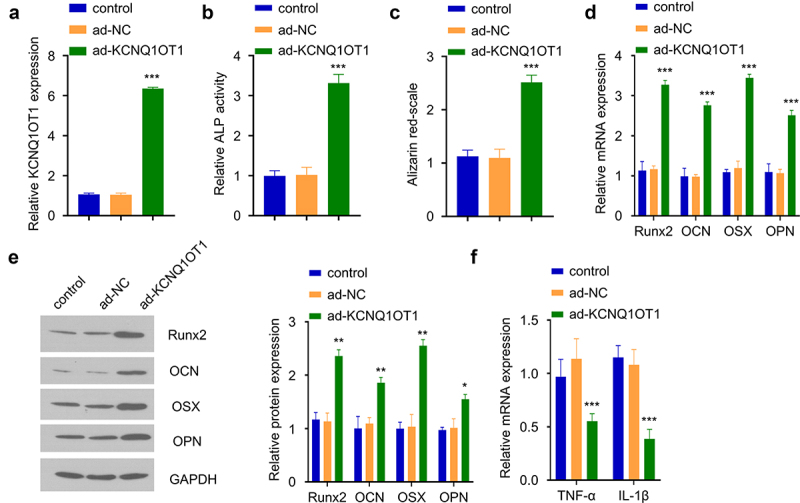
Overexpression of KCNQ1OT1 supports osteogenic differentiation in SpA-infected hBMSCs. (a) Expression of KCNQ1OT1 after transfection with ad-KCNQ1OT1. (b) ALP activity in SpA-treated HBMSCs after overexpressing KCNQ1OT1. (c) Alizarin red S staining in KCNQ1OT1-overexpressing hBMSCs under the treatment of SpA. (d-e) Levels of osteogenesis-related gene markers in different groups were measured by RT-qPCR (d) and Western blot assay (e). (f) Expressions of TNF-α and IL-1β in different groups was analyzed by RT-qPCR. * p < 0.05; **p < 0.01; ***p < 0.001.

To confirm the functional role of KCNQ1OT in osteogenic differentiation upon SpA-infection, we overexpressed KCNQ1OT1 in SpA-treated hBMSCs (0.5 µg/ml) by transfecting the cells with the ad-KCNQ1OT1 vector. qRT-PCR analysis showed that KCNQ1OT1 vector transfection strongly increased KCNQ1OT1 expression level as compared to the control or cells transfected with ad-NC vector (Figure 2(a)). We performed CCK-8 proliferation assay and the transfection of KCNQ1OT1 vector did not affect cellular proliferation in hBMSCs (Supplementary figure S2A). After osteogenic differentiation, ALP activity assay revealed that the ALP activity was strikingly increased in SpA-treated hBMSCs with KCNQ1OT1 overexpression (Figure 2(b)). The alizarin red S staining showed that KCNQ1OT1 overexpression in SpA-treated hBMSCs significantly enhanced cellular mineralization (Figure 2(c)). These effects were concomitant with an upregulation of osteogenic markers (Runx2, OCN, OSX, and OPN) at mRNA and protein levels upon KCNQ1OT1 overexpression (Figures 2(d,e)). We also found that overexpressing KCNQ1OT1 attenuated the expression of inflammatory cytokines TNF-α and IL-1β (Figure 2(f)). The above data indicate that KCNQ1OT1 overexpression supports osteogenic differentiation in SpA-infected hBMSCs.

### Knockdown of miR-29b-3p abrogates the inhibition of SpA-infection on osteogenic differentiation

**Figure 3.**
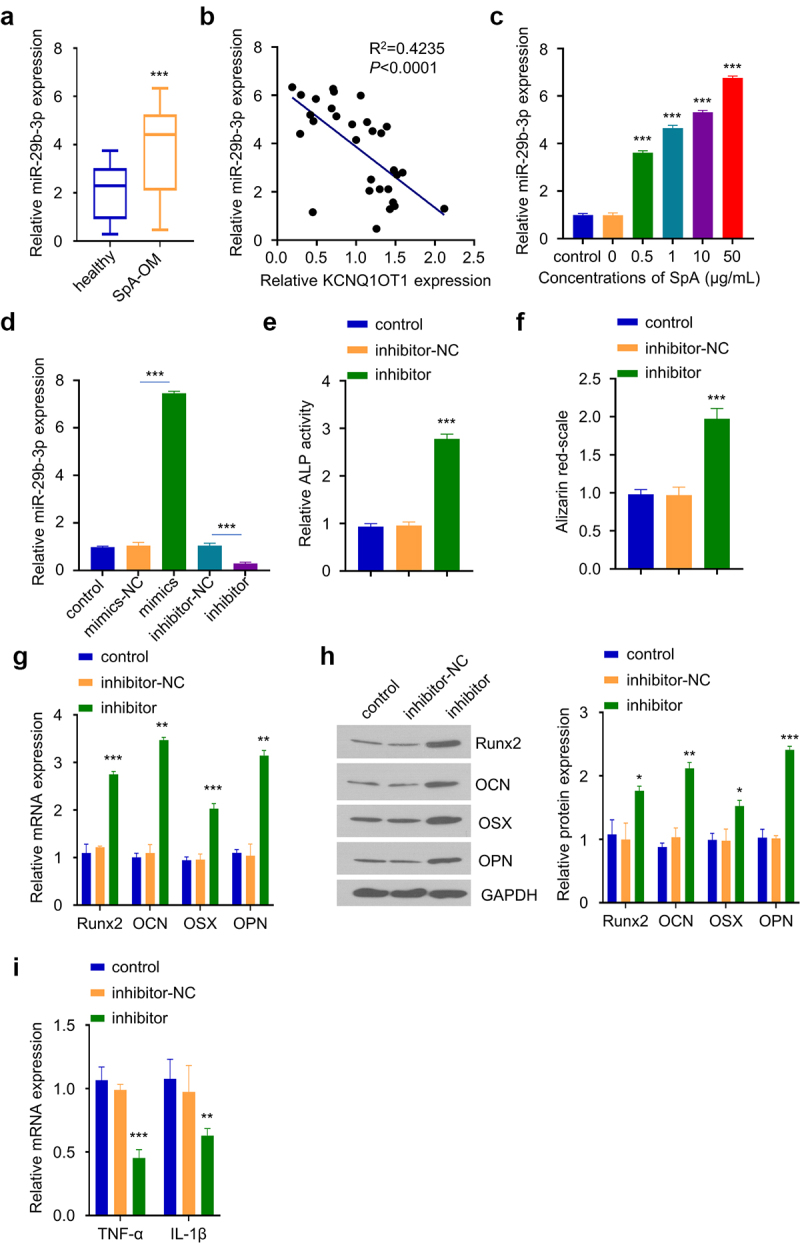
Silencing of miR-29b-3p improves osteogenic differentiation in SpA-infected hBMSCs. (a) Expression of miR-29b-3p in serum samples from OM patients and healthy controls. (b) Correlation between KCNQ1OT1 and miR-29b-3p expression level in the serum samples of OM patients. (c) Expressions of miR-29b-3p in SpA-treated hBMSCs was analyzed by RT-qPCR. (d) The expression of miR-29b-3p was analyzed after transfection with miR-29b-3p inhibitor or mimic. (e) ALP activity assay in SpA-treated hBMSCs after the transfection with miR-29b-3p inhibitor. (f) Alizarin red S staining in SpA-treated hBMSCs after the transfection with miR-29b-3p inhibitor. (d-e) Expression of osteogenic markers in SpA-treated hBMSCs after the transfection with miR-29b-3p inhibitor were examined by RT-qPCR (g) and Western blot (h). (i) Expression of TNF-α and IL-1β in SpA-treated hBMSCs after the transfection with miR-29b-3p inhibitor. *p < 0.05; **p < 0.01; ***p < 0.001.

In order to confirm the role of miR-29b-3p in OM progression, we first compared the expression levels of miR-29b-3p in serum samples of OM patients and healthy controls. qRT-PCR analysis revealed a significant increase of miR-29b-3p expression in OM patients (Figure 3(a)). Furthermore, miR-29b-3p expression level was negatively correlated with KCNQ1OT1 level in OM serum samples (Figure 3(b)). In addition, the infection of SpA also upregulated miR-29b-3p expression in a dose-dependent manner in hBMSCs. (Figure 3(c)). We therefore hypothesized that miR-29b-3p may mediate the inhibitory effect of SpA-infection. To test this hypothesis, we transfected hBMSCs with miR-29b-3p inhibitor, which could significantly suppress the expression of miR-29b-3p (Figure 3(d)). ALP activity assay revealed that ALP activity was strikingly increased in SpA-treated hBMSCs in the presence of miR-29b-3p inhibitor (Figure 3(e)). Similarly, after decreasing miR-29b-3p expression in SpA-treated hBMSCs, alizarin red staining was significantly enhanced (Figure 3(f)), which was accompanied with the upregulation of osteogenic markers (Runx2, OCN, OSX, and OPN) (Figures 3(g,h)). In contrast, the expression of TNF-α and IL-1β expressions was suppressed (Figure 3(i)). The above data indicate that miR-29b-3p is a negative regulator, which undermines osteogenic differentiation in SpA-infected hBMSCs.

### KCNQ1OT1 sponged miR-29b-3p

**Figure 4.**
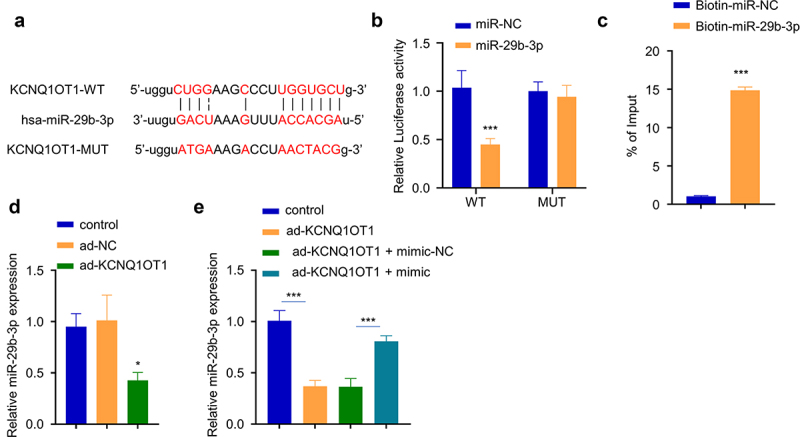
KCNQ1OT1 interacts with miR-29b-3p. (a) The potential binding sequence between KCNQ1OT and miR-29b-3p was predicted by Starbase database. (b) Dual-luciferase reporter assay using WT and MUT reporter in the presence of miR-29b-3p mimic or miR-NC. (c) RNA pull-down assay using biotin-labeled miR-NC or miR-29b-3p. (d) miR-29b-3p expression after KCNQ1OT1 overexpression. (e) miR-29b-3p expression level after co-transfection with ad-KCNQ1OT and miR-29b-3 mimic. * p < 0.05; **p < 0.01; ***p < 0.001.

Since the miR-29b-3p expression level was negatively correlated with KCNQ1OT1 level in OM serum, we used online Starbase to predict that there was a potential binding sequence between KCNQ1OT1 and miR-29b-3p (Figure 4(a)). To confirm their functional interactions, we performed dual-luciferase reporter assay using KCNQ1OT1-WT and KCNQ1OT1-MUT reporter in the presence of miR-29b-3p mimic or miR-NC. The results showed that the activity of KCNQ1OT1-WT reporter was significantly decreased by miR-29b-3p mimic, while no effect was observed in KCNQ1OT1-mut reporter containing mutated binding site (Figure 4(b)). Similarly, RNA pull-down assay demonstrated that biotin-labeled miR-29b-3p significantly enriched KCNQ1OT1 (Figure 4(c)), suggesting the direct interaction between KCNQ1OT1 and miR-29b-3p. KCNQ1OT1 overexpression in hBMSCs caused a significant reduction of miR-29b-3p level (Figure 4(d)), which could be partially reversed by co-transfection with miR-29b-3p mimic (Figure 4(e)). Together, these data suggest that KCNQ1OT1 targets miR-29b-3p to regulate its expression.

### miR-29b-3p is a downstream regulator of KCNQ1OT1 biological function in hBMSCs osteogenic differentiation

**Figure 5.**
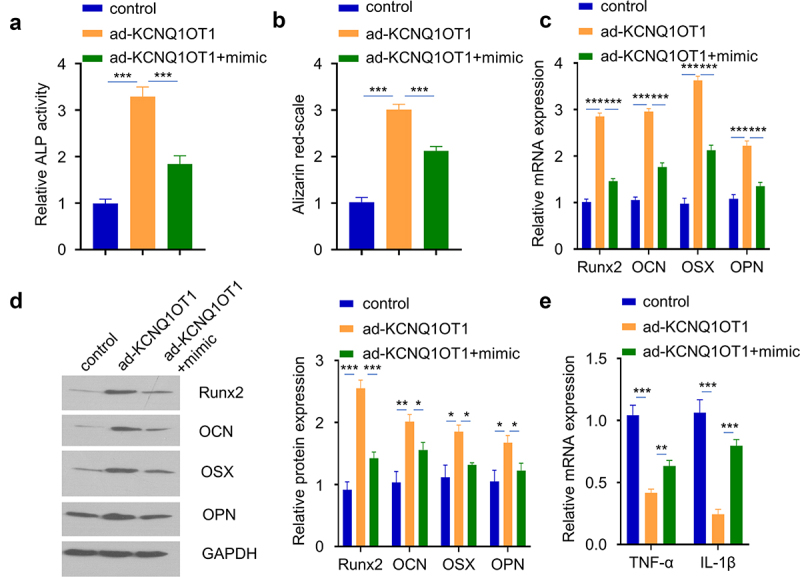
miR-29b-3p mediates the effect of KCNQ1OT1. hBMSCs were transfected with ad-KCNQ1OT1, or ad-KCNQ1OT1+ miR-29b-3 mimic. (a) ALP activity was measured in SpA-treated hBMSCs in different groups. (b) Alizarin red S staining in SpA-treated hBMSCs of different groups. (c-d) Expressions of osteogenic markers in different groups were examined by RT-qPCR (c) and Western blot assay (d). (e) TNF-α and IL-1β levels were analyzed by RT-qPCR in different groups. * p < 0.05; **p < 0.01; ***p < 0.001.

Since miR-29b-3p is a downstream target of KCNQ1OT1, and miR-29b-3p regulates osteogenic differentiation. We next sought to investigate whether miR-29b-3p mediates KCNQ1OT1 biological function in hBMSCs osteogenic differentiation. hBMSCs with KCNQ1OT1 overexpression was co-transfected with miR-29b-3p mimic. The transfection of KCNQ1OT1 expression vector and miR-29b-3p mimic did not affect cell proliferation (Supplementary figure 2B). However, the effect of KCNQ1OT1 in promoting osteogenic differentiation in SpA-infected hBMSCs was significantly attenuated by the co-transfection of miR-29b-3p mimic, as manifested by the reduced ALP activity (Figure 5(a)), diminished alizarin red S accumulation (Figure 5(b)), as well as the impaired upregulation levels of osteogenic markers (Runx2, OCN, and OPN) (Figures 5(c,d)). In the meanwhile, the suppressive effect of KCNQ1OT1 overexpression on inflammatory cytokines was also impaired by miR-29b-3p mimic (Figure 5(e)). Therefore, we conclude that miR-29b-3p is a downstream effector of KCNQ1OT1 biological function in hBMSCs osteogenic differentiation.

## Discussion

OM is a skeletal disease caused by the infection and invasion of microbial pathogens, which leads to progressive bone destruction [17]. The pathological changes in OM are mainly characterized by bone destruction and bone defects and are always associated with the imbalance between osteoblasts and osteoclasts during bone remodeling [18]. hBMSCs play pivotal roles in bone tissue renewal and are the main source of bone progenitor cells [19]. hBMSCs possess the ability of osteogenic differentiation by generating osteoblasts [20]. SpA has been recognized as the most common causative agent in OM, which could impair osteogenic differentiation of hBMSCs [21]. In the present study, we used SpA to simulate the microbial infection of hBMSCs in OM. Increasing the dose of SpA infection significantly impairs osteogenic differentiation of hBMSCs, as manifested by the reduced ALP activity, alizarin red S staining accumulation, as well as the downregulation of osteogenic markers. Together, these data imply that SpA infection significantly suppress the process of osteogenic differentiation in hBMSCs.

LncRNAs can act as competitive endogenous RNAs to regulate gene expression [22]. In recent years, lncRNAs have been implicated in the development and progression of various skeletal diseases, including osteoporosis and OM. For instance, lncRNA MSC-AS1 promotes osteogenic differentiation by regulating bone morphogenic protein 2 (BMP2) expression through targeting miRNA-140-5p, thereby attenuating osteoporosis [23]. A novel lncRNA GAS5 could alleviate osteoporosis progression by suppressing the miR-498/RUN2 axis [24]. LncRNA FAM83 H-AS1 was reported to be downregulated in SpA-treated hBMSCs, and it regulates osteogenic differentiation through the miR-541-3p/WNT3A pathway [25]. The increased level of inflammatory factors could also contribute to the development of OM [7]. A previous study found that KCNQ1OT1 suppresses the inflammation of vascular smooth muscle cells through IκBa in intimal hyperplasia [26]. Other studies also demonstrated that KCNQ1OT1 is significantly upregulated in hBMSCs during osteogenic differentiation [27–30]. In our work, we reported that KCNQ1OT1 is downregulated in OM samples and SpA-infected hBMSCs. Furthermore, KCNQ1OT1 overexpression promotes osteogenic differentiation and ameliorates the inflammation in SpA-treated hBMSCs. Collectively, our results suggest that KCNQ1OT1 supports osteogenic differentiation.

MicroRNAs (miRNAs) are small non-coding RNAs with a length of 18 ~ 22 nucleotides [31], which post-transcriptionally regulate gene expression by binding to the 3ʹUTR region of target mRNAs [32,33]. Meanwhile, miRNAs are involved in biological processes such as cell apoptosis, proliferation, differentiation, and the pathogenesis of many diseases [34–36]. A growing number of miRNAs have been implicated in the osteogenic differentiation of hBMSCs. For instance, Zhang et al. [37] reported that miR-10a-5p could inhibit hBMSCs osteogenic differentiation, which may impair bone repair progress. Zha et al. [38] showed that miR-920 is downregulated in osteoporosis, and miR-920 could promote osteogenic differentiation by targeting HOXA7. miR-29b-3p inhibits osteogenic differentiation in hBMSCs and aggravate bone formation [39]. In the present study, we found that miR-29b-3p is highly expressed in OM patient serum and SpA-induced hBMSCs. miR-29b-3p knockdown can alleviate the inflammatory response and enhance the osteogenic differentiation in SpA-induced hBMSCs. Importantly, we identified the binding site between KCNQ1OT1 and miR-29b-3p, and demonstrated that KCNQ1OT1 negatively regulates miR-29b-3p. We further showed that miR-29b-3p is a downstream regulator of KCNQ1OT1 biological function in hBMSCs osteogenic differentiation. Our results suggest that KCNQ1OT1/miR-29b-3p axis may regulate the progression of OM due to SpA infection.

Despite these novel findings in our study, there are some limitations worth mentioning. First, osteogenesis and bone resorption interactions are complex biological processes, and both osteoblast and osteoclast activities should be further investigated in OM model. In addition, the regulatory mechanism by which KCNQ1OT1 is downregulated upon SpA infection in OM needs to be elucidated. Furthermore, the downstream genes that participate in KCNQ1OT1/miR-29b-3p regulatory axis need to be clarified.

## Conclusion

In summary, our study showed that KCNQ1OT1 could attenuate inflammatory response and support osteogenic differentiation in hBMSCs upon SpA infection. miR-29b-3p is a downstream regulator of KCNQ1OT1 biological function in the osteogenic differentiation of hBMSCs. Given the observation that KCNQ1OT1 is downregulated, while miR-29b-3p is upregulated in the serum samples of OM patients with SpA infection, KCNQ1OT1/miR-29b-3p regulatory axis may contribute to the progression of OM.

## Supplementary Material

Supplemental Material

## Data Availability

The data during the current study are available from the corresponding author on reasonable request.

## Highlights

- KCNQ1OT1 is downregulated, while miR-29b-3p is upregulated in the serum of OM patients.
- SpA infection downregulates KCNQ1OT1 and impairs osteogenic differentiation.
- KCNQ1OT1 supports osteogenic differentiation by sponging miR-29b-3p.
