# Supplementary figures and images for: Long non-coding RNA KCNQ1OT1 overexpression promotes osteogenic differentiation of staphylococcus aureus-infected human bone mesenchymal stem cells by sponging microRNA miR-29b-3p
Source: Bioengineered. 2022 Feb 28;13(3):5855–67. doi: 10.1080/21655979.2022.2037898 (PMC8973675; doi:10.1080/21655979.2022.2037898)

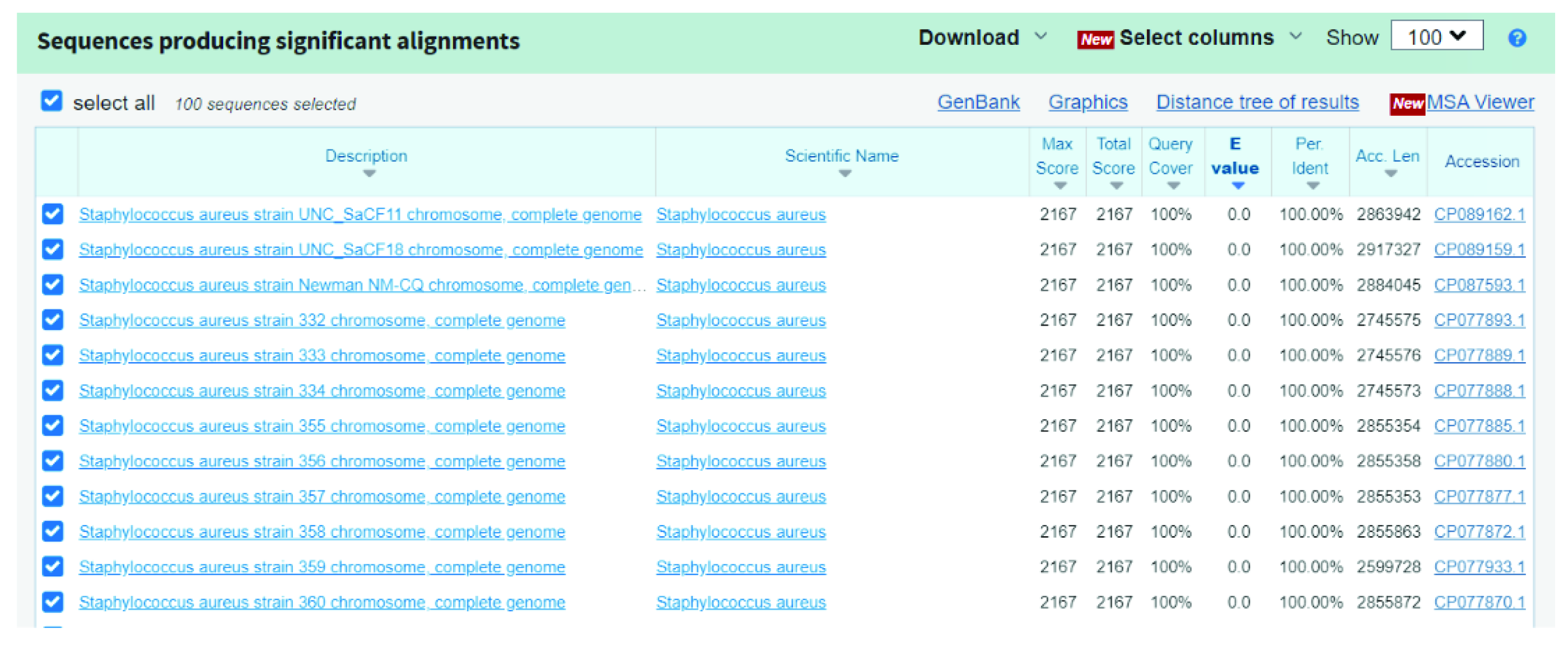

Supplement: Supplemental Material [file KBIE_A_2037898_SM6671.zip › supplementary/Figure S1.tif]

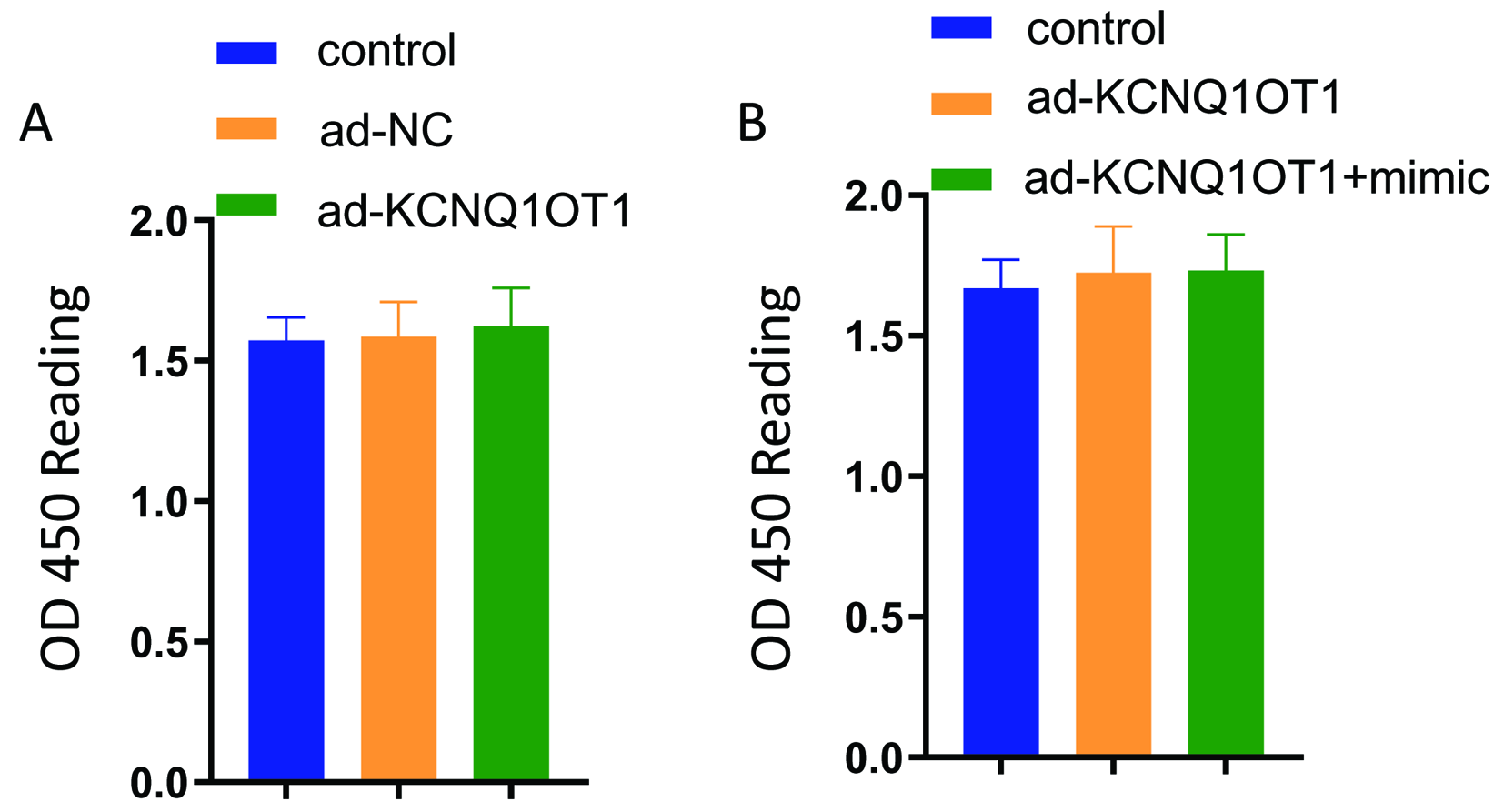

Supplement: Supplemental Material [file KBIE_A_2037898_SM6671.zip › supplementary/Figure S2.tif]
